# Lymphocyte-to-Monocyte Ratio and All-Cause Mortality in Populations With Abdominal Aortic Calcification: A Prospective Cohort Study

**DOI:** 10.1155/mi/9358261

**Published:** 2025-07-12

**Authors:** Jingjing Huang, Chunyong Chen

**Affiliations:** ^1^Cardiac Intensive Care Unit, The First Affiliated Hospital of Guangxi Medical University, Nanning, Guangxi, China; ^2^Department of Neurology, The First Affiliated Hospital of Guangxi Medical University, Nanning, Guangxi, China

**Keywords:** abdominal aortic calcification, all-cause mortality, lymphocyte-to-monocyte ratio, National Health and Nutritional Examination Surveys (NHANES), prospective cohort

## Abstract

**Objective:** The identification of reliable prognostic markers is essential for the effective management of abdominal aortic calcification (AAC). This research focused on assessing whether the lymphocyte-to-monocyte ratio (LMR) correlates with long-term mortality risk in the AAC population.

**Methods:** This analysis included 888 adults with AAC from National Health and Nutrition Examination Survey (NHANES) 2013–2014. Mortality risk was assessed using Cox proportional hazards models and Kaplan–Meier curves. Nonlinear associations between the LMR and mortality were examined with restricted cubic spline (RCS). The predictive ability was evaluated by time-dependent receiver operating characteristic (ROC) analysis.

**Results:** Over median follow-up for 71 months, 145 deaths were recorded. After adjusting for covariates, higher LMR was found to be significantly associated with a reduced risk of all-cause mortality, with a 28% decrease in risk per one-unit increment in LMR (hazard ratio; HR = 0.72, 95% confidence interval (CI): 0.57–0.91, *p*=0.02). This was consistent across quartiles. A nonlinear relationship was noted; below LMR 4.49, risk decreased (HR = 0.49, 95% CI: 0.40–0.60, *p* < 0.0001); above it, LMR was not significantly linked to mortality (HR = 1.14, 95% CI: 0.77–1.7, *p*=0.51). The area under the curve (AUC) for 2-, 4-, and 6-year survival were 0.647, 0.707, and 0.682, respectively.

**Conclusions:** Higher LMR is significantly associated with lower all-cause mortality in individuals with AAC, suggesting its potential utility as a prognostic marker in this population.

**Summary**

• The findings support the potential utility of lymphocyte-to-monocyte ratio (LMR) as a prognostic marker in managing patients with abdominal aortic calcification (AAC). Clinicians could use LMR to stratify patients' mortality risk and tailor interventions accordingly.
• This study reveals a previously unreported association between higher LMR and significantly reduced risk of all-cause mortality in patients with AAC.
• The research highlights a nonlinear relationship between LMR and all-cause mortality, identifying a threshold value (LMR of 4.49). Below this threshold, increased LMR strongly correlates with lower mortality risk, whereas above it, the association is not significant.

## 1. Introduction

Abdominal aortic calcification (AAC) has been presented throughout history and is increasingly recognized as an independent contributor to cardiovascular disease (CVD) [1]. Additionally, AAC serves as a significant marker of systemic atherosclerosis and is linked to higher cardiovascular morbidity and mortality [2–4]. AAC reflects a chronic inflammatory state and is often observed in conjunction with traditional cardiovascular risk factors such as hypertension [5], coronary heart disease (CHD) [5, 6], kidney disease [2, 7], and diabetes [8]. Increased occurrences of AAC were linked to older age, smoking, and hypertension [9]. The severity of AAC in hemodialysis patients was related to systolic blood pressure levels and the serum calcium-phosphate product [10]. Despite its clinical relevance, the identification of reliable prognostic biomarkers that can effectively guide the management of patients with AAC remains a challenge.

Recently, the lymphocyte-to-monocyte ratio (LMR) has garnered attention as a potential inflammatory marker that could provide prognostic insights into various cardiovascular conditions [11, 12]. Lymphocytes and monocytes are key components of the immune system, and their relative proportions may reflect underlying inflammatory processes that contribute to CVD progression and mortality [13, 14]. Previous studies have demonstrated that a higher LMR is associated with better prognosis in CHD, while a lower LMR correlates with increased inflammatory burden and worse outcomes in 1 year follow-up [15]. Despite extensive research on the prognostic value of LMR in CVDs, there is a significant gap in our understanding of its relationship with AAC-related mortality, necessitating further investigation to elucidate this association.

This study aimed to explore the association between LMR and all-cause mortality in adults with AAC, as previous research in this area remains lacking. This investigation provides important insights into the potential of LMR as a prognostic marker in patients with AAC, highlighting the significance of inflammatory markers in cardiovascular risk stratification. Such information is critical for clinicians aiming to identify high-risk patients who may benefit from more intensive management strategies. Given the progressive nature of aortic calcification and its impact on patient outcomes, incorporating reliable biomarkers like LMR into clinical practice could enhance prognostic accuracy and therapeutic decision-making.

## 2. Methods

### 2.1. Study Population

National Health and Nutrition Examination Survey (NHANES) provides a cross-sectional dataset designed to reflect the health and nutritional characteristics of the United States population. The application of sample weights accommodates the complex sampling framework, permitting estimates that are generalizable to national demographic distributions across age, sex, and ethnicity as outlined in national census definitions [16]. Information was obtained through standardized home interviews, physical examinations conducted at mobile centers, and laboratory assessments, all carried out through a multistage probability sampling method. The NHANES protocol received approval from the National Center for Health Statistics (NCHS) ethics review board, with written informed consent obtained from all participants.

**Figure 1:**
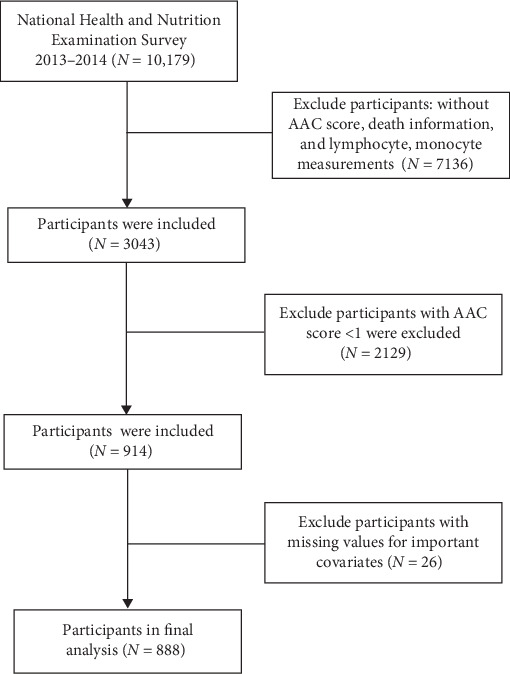
The study flowchart. AAC, abdominal aortic calcification.

An initial pool of 10,179 subjects from the 2013–2014 NHANES cycle was considered. Based on predefined exclusion criteria—including absence of AAC scores, mortality data, or inflammatory markers (*n* = 7136), AAC score less than 1 (*n* = 2129), and missing essential covariate data (*n* = 26)—a total of 888 eligible participants remained for analysis. The detailed data selection process is illustrated in Figure 1. The data employed in this study is publicly accessible (https://wwwn.cdc.gov/nchs/nhanes/default.aspx) and has been demographically weighted for subsequent analyses.

### 2.2. Measurement of LMR

According to the NHANES protocol, lymphocyte and monocyte counts were determined through a complete blood count utilizing automated hematology analyzers (specifically the Colter DxH 800 analyzer) and were expressed in units of × 10^3^ cells/µL. The LMR was derived by dividing the absolute count of lymphocytes by the absolute count of monocytes.

### 2.3. Measurement and Definition of AAC

Dual-energy X-ray absorptiometry (DXA) using the Discovery A densitometer (Hologic, Marlborough, MA, USA) was employed to scan the lumbar spine (vertebrae L1–L4), applying the Kauppila scoring system to assess and quantify AAC. Certified and trained radiologic technologists performed the DXA scans at the NHANES mobile examination centers. In this analysis, Kauppila scores ranged from 0 to 24 (AAC total 24 score), with scores of ≥1 signifying the presence of calcification, thus, identifying AAC. Further details on AAC measurement procedures can be found at https://www.cdc.gov/Nchs/Nhanes/20132014/DXXAAC_H.html.

### 2.4. Ascertainment of Mortality and Follow-Up

Mortality status was ascertained by linking NHANES data with the National Death Index (NDI) records, which can be accessed at https://www.cdc.gov/nchs/data-linkage/mortality-public.htm. Participants were classified as either deceased or alive based on NDI data. The follow-up period was determined by calculating the time from the date of the NHANES examination to the date of death or December 31, 2019, whichever came first.

### 2.5. Assessment of Covariates

Collected covariates encompassed demographic details (such as age, sex, race/ethnicity, and education), lifestyle factors (smoking and alcohol consumption), clinical and biochemical parameters (body mass index (BMI), poverty income ratio (PIR), hemoglobin A1c (HbA1c), low-density lipoprotein cholesterol (LDL-C), total cholesterol (TC), creatinine, and serum uric acid), as well as disease history (diabetes, hypertension, CHD, heart failure, and stroke). Race was categorized as non-hispanic white, non-hispanic black, mexican american, other hispanic, and other/multiracial. Education levels were classified into “Less than 9th Grade,” “9–11th Grade,” “ = High School Grad/GED,” “Some College or Associate Degree,” and “College Graduate or above” [17]. Smoking status was divided into never smokers (individuals who smoked fewer than 100 cigarets in their lifetime), former smokers (those who smoked 100 or more cigarets in their lifetime but were not currently smoking), and current smokers (individuals who smoked cigarets daily or on some days at the time of the survey). BMI was computed as weight in kilograms divided by the square of height in meters and categorized as normal (<25.0 kg/m^2^), overweight (25.0–29.9 kg/m^2^), and obese (≥30.0 kg/m^2^). DM was identified as either undergoing treatment for or having a medical diagnosis of hyperglycemia, with hemoglobin A1c levels of ≥6.5%, fasting blood glucose levels of ≥126 mg/dL, or a 2-h blood glucose level of ≥200 mg/dL. Hypertension was defined as the use of antihypertensive medications, a medical diagnosis of hypertension, or three consecutive measurements of systolic blood pressure of ≥140 mmHg or diastolic blood pressure of ≥90 mmHg.

### 2.6. Statistical Analysis

Recognizing that NHANES utilizes a complex, multistage, probability sampling design to select representative participants, we incorporated sample weights, clustering, and stratification into all analyses to achieve national estimates. Continuous variables were presented as weighted means ± standard error, while categorical variables were expressed as numbers (*n*) with percentages. One-way ANOVA, Kruskal–Wallis *H* test, or Chi-squared test were used to compare continuous and categorical variables across different groups, respectively.

To examine the associations between the LMR and the risk of all-cause mortality, we utilized a multivariate Cox regression model to estimate hazard ratios (HRs) and 95% confidence intervals (CIs). Given that LMR distribution is right-skewed, values were divided into quartiles, analyzed both as continuous and categorical variables. The first quartile (Q1) served as the reference group, with median values assigned to each category to assess linear trends. Variables with *p* < 0.1 in univariate analysis or those clinically relevant to prognosis were included in the multivariable Cox regression model. We developed three models: The first model was unadjusted; the second was adjusted for age, sex, and race; and the fully adjusted model additionally included education level, PIR, BMI, smoking status, alcohol consumption, HbA1c, creatinine, serum uric acid, LDL-C, DM, stroke, hypertension, CHF, CHD, and AAC total 24 score. An AAC score >6 was defined as “severe AAC” [18, 19]. Kaplan–Meier survival analysis was used to compare survival probabilities among different LMR levels in individuals with AAC, with log-rank tests employed for group comparisons.

Stratified analyses were also conducted by gender (male and female), age group (≥65 and <65), smoking status (no and yes), alcohol consumption (no and yes), DM (no and yes), CHD (no and yes), stroke (no and yes), heart failure (no and yes), and hypertension (no and yes). Possible interactions between these stratification factors and LMR were also assessed. Additionally, restricted cubic spline (RCS) and threshold effect analysis were utilized to evaluate any nonlinear relationships between LMR and all-cause mortality.

*p*-value < 0.05 was deemed statistically significant. Statistical analyses were performed utilizing R software (version 4.2.1).

## 3. Results

### 3.1. Baseline Characteristics of Study Participants

A total of 888 individuals were included in the study, with 49.9% female participants and an average age of 64.83 ± 11.9 years. During a median follow-up period of 71 months (interquartile range (IQR), 64.0–78.0 months), the incidence of all-cause mortality among the participants was 16.33%. The weighted mean ± standard error of the natural LMR was 3.63 ± 1.49.  Table 1 presented the weighted sociodemographic and medical characteristics of participants across the LMR quartiles. Participants in the lower LMR quartiles tended to be older, more frequently female, and more likely to be smokers with comorbid hypertension and heart failure. They also exhibited higher values in waist measurement, creatinine, TC, low-density lipoprotein (LDL), and AAC scores (all *p* < 0.05). Supporting Information 1: Table S1 provided baseline characteristics segmented by all-cause mortality.

### 3.2. Association Between LMR and All-Cause Mortality in US Adults With AAC

**Figure 2:**
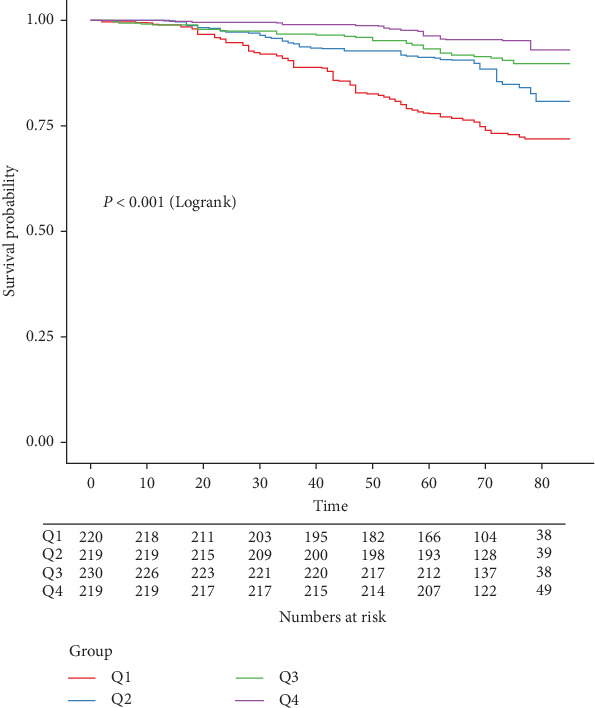
Weighted Kaplan–Meier curve of the survival rate with LMR quantile. The Kaplan–Meier curves demonstrated that individuals with AAC in the lowest quartile of the LMR group had significantly lower all-cause survival probabilities compared to those in the highest quartile group (*p* < 0.001). AAC, abdominal aortic calcification; LMR, lymphocyte-to-monocyte ratio.

During a median follow-up period of 71 months (IQR, 64.0–78.0 months), 145 (16.3%) of the 888 participants passed away. As shown in Table 2, the LMR was significantly associated with a reduced risk of all-cause mortality in the unadjusted model (HR = 0.57, 95% CI: 0.47–0.71). This association remained strong and statistically significant after multivariable adjustments, with Model 2 showing an HR of 0.69 (95% CI: 0.59–0.81) and Model 3 showing an HR of 0.72 (95% CI: 0.57–0.91). Compared to the first quartile of LMR, the HRs for participants in the third and fourth quartiles were consistently lower across all models. In Model 1, the HRs were 0.32 (95% CI: 0.18–0.56) for the third quartile and 0.18 (95% CI: 0.09–0.33) for the fourth quartile, with a significant trend (*p* for trend <0.001). In Model 2, the HRs were 0.43 (95% CI: 0.24–0.77) for the third quartile and 0.34 (95% CI: 0.17–0.66) for the fourth quartile, also showing a significant trend (*p* for trend <0.001). Model 3 presented HRs of 0.40 (95% CI: 0.19–0.55) for the third quartile and 0.34 (95% CI: 0.13–0.90) for the fourth quartile (*p* for trend = 0.02). The Kaplan–Meier survival curves illustrated significantly lower all-cause survival probabilities for individuals in the lower quartile levels of LMR compared to those in the higher quartiles (*p* < 0.001) (Figure 2).

Subgroup analyses and interaction tests were conducted to examine the robustness of the relationship between LMR and all-cause mortality across various subgroups, including age, sex, smoking status, alcohol consumption, obesity, DM, hypertension, and history of CHD, heart failure, and stroke. The results indicated a consistent association without significant interactions between these characteristics and LMR (*p* for interaction >0.05). Lastly, subgroup analysis by severity of AAC showed no significant interaction (*p* for interaction = 0.71) (Table 3).

### 3.3. Nonlinear Relationship Between LMR and All-Cause Mortality in US Adults With AAC

**Figure 3:**
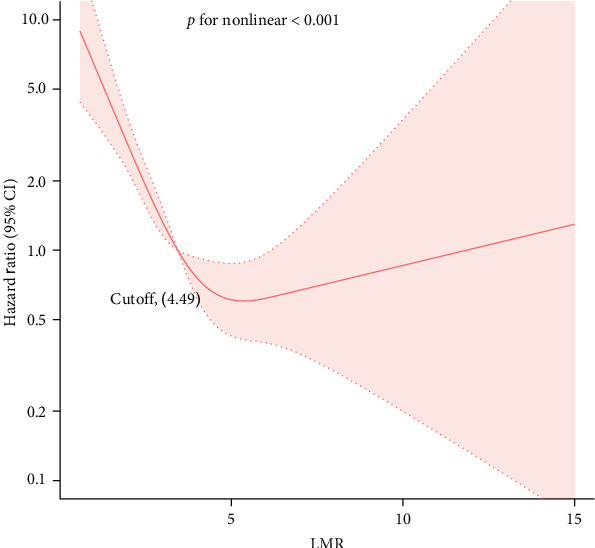
Restricted cubic spline to evaluate the nonlinear relationship between LMR and the all-cause mortality in US adults with AAC. Adjustment for age, sex, race, education, BMI, PIR, smoking status, drink consumption, HbA1c, Cr, serum uric acid, LDL-C, DM, HTN, CHD, HF, stroke, and AAC score. The blue solid line represents the probability of all-cause mortality, the yellow and purple dotted line represents the 95% confidence interval. AAC, abdominal aortic calcification; BMI, body mass index; CHD, coronary heart disease; CI, confidence interval; Cr, creatinine; DM, diabetes mellitus; HbA1c, hemoglobin A1c; HF, heart failure; HR, hazard ratio; HTN, hypertension; LDL-C, low-density lipoprotein cholesterol; LMR, lymphocyte-to-monocyte ratio; PIR, family poverty income ratio.

In this study, we employed the RCS to explore potential nonlinear relationships between the LMR and all-cause mortality in US adults with AAC. The fully adjusted model revealed a nonlinear relationship between LMR and all-cause mortality (Figure 3).

Threshold effect analysis indicated that LMR was associated with all-cause mortality in a nonlinear fashion when multiple confounders were adjusted for, with a threshold value identified at an LMR of 4.49). Below this threshold, higher LMR was significantly associated with a decreased risk of all-cause mortality (HR = 0.49, 95% CI: 0.40–0.60, *p* < 0.0001). However, above this threshold, LMR did not show a significant association with all-cause mortality (HR = 1.14, 95% CI: 0.77–1.7, *p*=0.51; Table 4).

### 3.4. The Predictive Ability of LMR for All-Cause in US Adults With AAC

**Figure 4:**
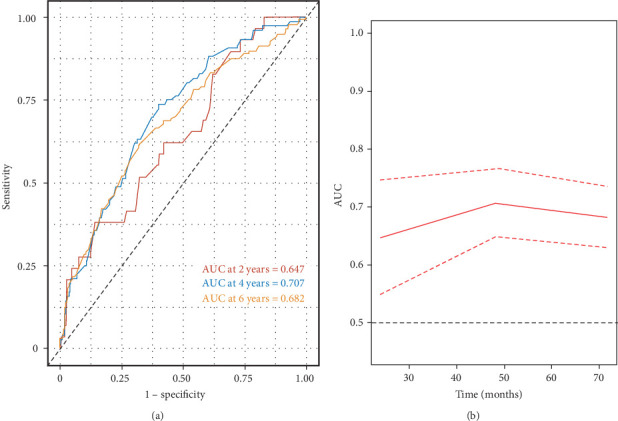
Time-dependent ROC curves (A) and time-dependent AUC values (B) (with 95% CI) of the LMR for predicting all-cause mortality in US adults with AAC. AAC, abdominal aortic calcification; AUC, area under the curve; CI, confidence interval; LMR, lymphocyte-to-monocyte ratio; ROC, receiver operating characteristic.

The time-dependent receiver operating characteristic (ROC) curve analysis showed that the area under the curve (AUC) for the LMR was 0.647, 0.707, and 0.682 for predicting all-cause mortality at 2 years, 4 years, and 6 years, respectively (Figure 4). These findings indicate that the predictive ability of LMR for all-cause mortality remains consistently effective across different time intervals. Moreover, the results suggest that the predictive value of LMR for all-cause mortality is superior to that of using lymphocyte or monocyte counts alone over 2-year, 4-year, and 6-year periods (Supporting Information 2: Figure S1).

## 4. Discussion

In this study, we included 888 American adults with a mean age of 64.83 years and observed an all-cause mortality rate of 16.33% over a median follow-up period of 71 months (IQR, 64.0–78.0 months). After multivariate adjustment, we found that LMR was significantly and independently associated with a lower risk of all-cause mortality in populations with AAC. Furthermore, LMR demonstrated an effective predictive ability for all-cause mortality over 2, 4, and 6 years, compared to lymphocyte and monocytes alone. These findings remained consistent across various sensitivity and stratified analyses. Additionally, we also found a nonlinear relationship between LMR and all-cause mortality, and a threshold effect at the critical point 4.49. Below this threshold, higher LMR was significantly inversely associated with mortality risk, while above this threshold, the association was not statistically significant.

Previous research indicating the importance of inflammatory markers in cardio-cerebrovascular disease prognosis [5, 15], and showing that LMR independently predicts overall mortality and CVD mortality in the general population [20]. Moreover, existing literatures had recognized that LMR as an important prognostic marker in coronary artery disease [11, 12], and AAC [21]. However, the role of LMR in predicting all-cause mortality in AAC populations is unclear. To the best of our knowledge, this study is the first to investigate the predictive value of LMR for all-cause mortality in AAC patients, providing critical insights into the role of inflammation in AAC and its related outcomes. Our results demonstrated that higher LMR was consistently associated with a lower risk of all-cause mortality in AAC participants statistically significant, even after adjusting for multiple confounders. Participants in the lower quartile of LMR had significantly poorer survival probabilities compared to those in higher quartiles, underscoring the value of LMR in stratifying risk among patients with AAC. Among adults in the United States, LMR was linked to elevated AAC scores and an increased likelihood of severe AAC, suggesting its potential to be a valuable predictor of AAC risk [21].

It is widely recognized that peripheral blood Mononuclear cells (PBMCs), which include lymphocytes and monocytes/macrophages, are the primary producers of proinflammatory cytokines like IL-6 and TNF-α [21]. The production and release of TNF-α in monocytes further speed up vascular calcification in uremia [22]. Studies have confirmed that inflammatory cytokines and inflammatory status can promote vascular calcification [23]. LMR represents the body's inflammatory and immune status, lower LMR is typically caused by a decrease in lymphocytes or an increase in monocytes, reflecting an immunosuppressive state and chronic inflammatory response [24]. Higher LMR indicates a favorable balance between lymphocytes and monocytes, may reflect a lower inflammatory burden and better overall prognosis [25, 26]. In the process of atherosclerosis, monocytes migrate to the site of injury and transform into macrophages, promoting the formation of inflammatory and arterial calcification plaques [27, 28]. The reduction in lymphocytes may diminish the regulation of anti-inflammatory responses, further exacerbating local and systemic inflammation [29, 30]. Our study found that low LMR is associated with higher risk of all-cause mortality in AAC population, indicating that chronic inflammation plays a central role in the pathogenesis of AAC, and LMR can reflect the severity of this process.

The clinical implications of these findings are significant. Incorporating LMR into routine clinical assessments could enhance risk stratification and guide the management of patients with AAC. Given AAC's association with cardiovascular morbidity and mortality [4], identifying high-risk patients through reliable biomarkers like LMR could facilitate targeted interventions and improve patient outcomes.

This study has several limitations that should be acknowledged. First, the observational design significantly constrains our ability to establish definitive causal relationships between LMR and all-cause mortality. Consequently, our findings are correlational and cannot determine if alterations in LMR directly affect mortality risk. Second, the analysis is based on data from the NHANES 2013–2014 cycle, which may not adequately reflect the current population demographics or capture advancements in healthcare practices since then. Additionally, due to the inclusion of AAC data solely from the NHANES 2013–2014 cycle, the relatively small number of deaths recorded during the follow-up limits our capacity to conduct more granular analyses of mortality causes, thus, restricting our understanding of the relationship between LMR and specific mortality outcomes, such as cardiovascular mortality. Furthermore, our findings are derived specifically from adults with AAC, raising concerns about their generalizability to broader populations or individuals without AAC. Lastly, the biological mechanisms underlying the association between LMR and all-cause mortality are not fully elucidated. Additional research is essential to explore the pathways through which LMR influences health outcomes, particularly its roles in inflammation and CVD. Nevertheless, this investigation offers distinct methodological advantages. The analysis of a nationally representative NHANES cohort strengthens the generalizability of our findings across the general US population not residing in institutions. Moreover, the substantial sample size and strict NHANES data collection protocols provide additional statistical power and reduce potential measurement biases, enhancing the validity of our conclusions.

## 5. Conclusion

In conclusion, this study underscores the prognostic potential of LMR in predicting all-cause mortality among adults with AAC. The consistent associations observed across various models and subgroups, along with the identification of a nonlinear relationship and the demonstrated predictive capacity of LMR, suggest that it holds promise as a valuable biomarker in clinical practice. Further research is warranted to explore the mechanistic pathways linking LMR to cardiovascular outcomes and to validate these findings in larger, more diverse cohorts.

## Figures and Tables

**Table 1:** Baseline characteristics of included participants according to the quartile of LMR.

Variables	Overall	Quartile 1	Quartile 2	Quartile 3	Quartile 4	*p*-Value
*N*	888	220	219	230	219	—
Age (year)	64.83 ± 11.9	69.95 ± 10.73	65.32 ± 12.11	64.14 ± 11.01	59.94 ± 11.63	<0.01
Sex (%)						
Female	443 (49.89)	79 (35.91)	101 (46.12)	124 (53.91)	139 (63.47)	<0.01
Male	445 (50.11)	141 (64.09)	118 (53.88)	106 (46.09)	80 (36.53)	—
Race (%)						
Mexican American	91 (10.25)	19 (8.64)	24 (10.96)	22 (9.57)	26 (11.87)	<0.01
Non-hispanic black	137 (15.43)	32 (14.55)	28 (12.79)	41 (17.83)	36 (16.44)	—
Non-hispanic white	482 (54.28)	148 (67.27)	126 (57.53)	117 (50.87)	91 (41.55)	—
Other hispanic	70 (7.88)	10 (4.55)	15 (6.85)	25 (10.87)	20 (9.13)	—
Other/multiracial	108 (12.16)	11 (5.00)	26 (11.87)	25 (10.87)	46 (21.00)	—
Education level (%)						
9–11th grade	122 (13.74)	27 (12.27)	31 (14.16)	35 (15.22)	29 (13.24)	0.56
≥College graduate	212 (23.87)	62 (28.18)	54 (24.66)	46 (20.00)	50 (22.83)	—
High school graduation/GED	224 (25.23)	49 (22.27)	54 (24.66)	67 (29.13)	54 (24.66)	—
Less than 9th grade	82 (9.23)	15 (6.82)	18 (8.22)	23 (10.00)	26 (11.87)	—
Some college/AA degree	248 (27.93)	67 (30.45)	62 (28.31)	59 (25.65)	60 (27.40)	—
Alcohol consumption (%)	610 (71.76)	176 (82.63)	154 (72.99)	153 (69.86)	127 (61.35)	0.25
Smoke status (%)						
Current smoker	183 (20.61)	38 (17.27)	41 (18.72)	52 (22.61)	52 (23.74)	0.03
Former smoker	298 (33.56)	94 (42.73)	70 (31.96)	74 (32.17)	60 (27.40)	—
Never smoker	407 (45.83)	88 (40.00)	108 (49.32)	104 (45.22)	107 (48.86)	—
PIR	2.6 ± 1.59	2.8 ± 1.58	2.59 ± 1.59	2.44 ± 1.6	2.57 ± 1.59	0.13
BMI	27.67 ± 4.8	27.33 ± 5.01	27.86 ± 4.86	28.03 ± 4.51	27.44 ± 4.83	0.36
Waist size (cm)	99.03 ± 12.11	100.31 ± 13.15	99.8 ± 11.66	99.5 ± 11.57	96.49 ± 11.73	<0.01
Platelet (10^3^/μL)	223.39 ± 57.66	216.62 ± 57.71	221.9 ± 61.91	221.75 ± 56.61	233.41 ± 53.21	0.02
Neutrophil (10^3^/μL)	4.33 ± 1.64	4.88 ± 1.95	4.46 ± 1.63	4.19 ± 1.48	3.8 ± 1.21	<0.01
Lymphocyte (10^3^/μL)	2.07 ± 0.82	1.43 ± 0.46	1.97 ± 0.55	2.18 ± 0.57	2.7 ± 1.02	<0.01
Monocyte (10^3^/μL)	0.61 ± 0.21	0.73 ± 0.27	0.66 ± 0.19	0.57 ± 0.15	0.49 ± 0.14	<0.01
WBC (10^3^/μL)	7.28 ± 2.07	7.28 ± 2.25	7.37 ± 2.11	7.2 ± 1.95	7.26 ± 1.99	0.86
Glucose (mg/dL)	115.18 ± 26.68	115.1 ± 29.6	114.91 ± 21.5	117.19 ± 32.65	113.4 ± 20.67	0.51
HbA1c (%)	6.03 ± 1.1	5.93 ± 0.91	5.99 ± 1.01	6.18 ± 1.37	6.03 ± 1.01	0.08
Serum creatinine (mmol/L)	89.33 ± 63.69	105.67 ± 111.53	86.91 ± 26.06	83.73 ± 24.11	81.22 ± 48.44	<0.01
Serum uric acid (mmol/L)	333.97 ± 86	347.79 ± 90.63	336.78 ± 88.32	332.21 ± 85.62	319.13 ± 76.91	0.01
Total cholesterol (mmol/L)	4.96 ± 1.12	4.67 ± 1.07	4.93 ± 1.11	5.1 ± 1.16	5.12 ± 1.09	<0.01
Triglycerides (mmol/L)	1.41 ± 0.6	1.36 ± 0.54	1.44 ± 0.62	1.4 ± 0.51	1.46 ± 0.71	0.30
LDL-C (mmol/L)	2.93 ± 0.7	2.76 ± 0.73	2.96 ± 0.74	2.96 ± 0.63	3.05 ± 0.69	<0.01
HDL-C (mmol/L)	1.37 ± 0.4	1.39 ± 0.43	1.31 ± 0.37	1.4 ± 0.45	1.35 ± 0.35	0.08
AAC total 24 score	5.43 ± 4.53	7.23 ± 5.37	5.12 ± 4.21	5.25 ± 4.43	4.14 ± 3.35	<0.01
Hypertension (%)	540 (60.81)	143 (65.00)	144 (65.75)	141 (61.30)	112 (51.14)	0.01
Diabetes (%)	181 (20.38)	50 (22.73)	40 (18.26)	51 (22.17)	40 (18.26)	0.49
Heart failure (%)	50 (5.65)	21 (9.63)	15 (6.88)	6 (2.61)	8 (3.65)	0.01
CHD (%)	92 (10.36)	42 (19.09)	21 (9.59)	16 (6.96)	13 (5.94)	<0.01
Stroke (%)	60 (6.76)	22 (10.00)	16 (7.31)	13 (5.65)	9 (4.11)	0.08
LMR	3.63 ± 1.49	2.02 ± 0.46	3.01 ± 0.23	3.86 ± 0.26	5.62 ± 1.28	<0.01
Death (%)	145 (16.33)	67 (30.45)	36 (16.44)	25 (10.87)	17 (7.76)	<0.01

Abbreviations: AAC, abdominal aortic calcification; CHD, coronary heart disease; HbA1c, glycohemoglobin; HDL-C, high-density lipoprotein cholesterol; LDL-C, low-density lipoprotein cholesterol; LMR, lymphocyte-to-monocyte ratio; PIR, family poverty income ratio; WBC, white blood cell.

**Table 2:** Weighted association between LMR and all-cause mortality in US adults with abdominal aortic calcification.

LMR	Model 1	Model 2	Model 3
Continues	0.57 (0.47, 0.71)	0.69 (0.59, 0.81)	0.72 (0.57, 0.91)
Quartile 1	reference	reference	reference
Quartile 2	0.51 (0.31, 0.84)	0.69 (0.42, 1.150)	0.67 (0.25, 1.81)
Quartile 3	0.32 (0.18, 0.56)	0.43 (0.24, 0.77)	0.40 (0.19, 0.55)
Quartile 4	0.18 (0.09, 0.33)	0.34 (0.17, 0.66)	0.34 (0.13, 0.90)
*p* for trend	<0.001	<0.001	0.02

*Note:* Model 1: No adjusted. Model 2: Adjusted for age, sex and race. Model 3: Adjusted for age, sex, race, education, BMI, PIR, smoking status, alcohol consumption, HbA1c, Cr, serum uric acid, LDL-C, DM, HTN, CHD, HF, stroke, and AAC score.

Abbreviations: AAC, abdominal aortic calcification; BMI, body mass index; CHD, coronary heart disease; CI, confidence interval; Cr, creatinine; DM, diabetes mellitus; HbA1c, hemoglobin A1c; HF, heart failure; HR, hazard ratio; HTN, hypertension; LDL-C, low-density lipoprotein cholesterol; LMR, lymphocyte-to-monocyte ratio; PIR, family poverty income ratio.

**Table 3:** Subgroup analyses of LMR and all-cause mortality in US adults with AAC, stratified by participant characteristics.

Variables	Count	Percent	HR (95% CI)	*p*-Value	*p* for interaction
Overall	888	100	0.72 (0.57, 0.91)	<0.001	—
Sex	—	—	—	—	0.78
Female	443	49.9	0.72 (0.35, 0.89)	0.04	—
Male	445	50.1	0.75 (0.43, 1.30)	0.30	—
Age group	—	—	—	—	0.32
<65 years	410	46.2	0.42 (0.23, 0.76)	0.004	—
≥65 years	478	53.8	0.80 (0.58, 1.11)	0.18	—
Smoke	—	—	—	—	0.23
No	407	45.8	0.86 (0.58, 1.28)	0.036	—
Yes	481	54.2	0.50 (0.34, 0.74)	<0.001	—
Drink group	—	—	—	—	0.23
Drinker	610	68.7	0.48 (0.29, 0.78)	0.003	—
Nondrinker	240	27.0	1.19 (0.84, 1.68)	0.33	—
Hypertension	—	—	—	—	0.29
No	348	39.2	0.62 (0.38, 0.98)	0.05	—
Yes	540	60.8	0.62 (0.42, 0.91)	0.01	—
Diabetes	—	—	—	—	0.51
No	707	79.6	0.74 (0.52, 1.07)	0.11	—
Yes	181	20.4	0.29 (0.07, 1.15)	0.08	—
Heart failure	—	—	—	—	0.48
No	835	94.0	0.58 (0.35, 0.76)	0.001	—
Yes	50	5.6	0.73 (0.53, 1.16)	0.56	—
CHD	—	—	—	—	0.21
No	796	89.6	0.68 (0.43, 1.08)	0.1	—
Yes	92	10.4	0.38 (0.13, 1.11)	0.08	—
Stroke	—	—	—	—	0.25
No	828	93.2	0.74 (0.51, 1.19)	0.46	—
Yes	60	6.8	0.43 (0.31, 0.62)	<0.001	—
Obesity	—	—	—	—	0.27
No	630	70.9	0.75 (0.57, 0.98)	0.04	—
Yes	258	29.1	0.41 (0.21, 0.78)	0.01	—
Severe AAC	—	—	—	—	0.71
No	570	64.2	0.64 (0.43, 0.95)	0.03	—
Yes	318	35.8	0.63 (0.31, 1.26)	0.19	—

Abbreviations: AAC, abdominal aortic calcification; CHD, coronary heart disease; CI, confidence interval; HR, hazard ratio; LMR, lymphocyte-to-monocyte ratio.

**Table 4:** Threshold effect analysis of LMR on all-cause mortality in US adults with AAC.

LMR	HR (95% CI)	*p*-Value
Inflection point	4.49	—
<Threshold effect	0.49 (0.40, 0.60)	<0.0001
>Threshold effect	1.14 (0.77, 1.7)	0.51
*p* for log-likelihood ratio test	<0.001	—

Abbreviations: 95% CI, 95% confidence interval; HR, hazard ratio.

## Data Availability

The data used in this study are publicly available and can be accessed through the following link: https://wwwn.cdc.gov/nchs/nhanes/default.aspx.

## Nomenclature

AAC: Abdominal aortic calcification
AUC: Area under the curve
BMI: Body mass index
CDC: Centers for Disease Control and Prevention
CHD: Coronary heart disease
CHF: Heart failure
CI: Confidence interval
CVD: Cardiovascular disease
DM: Diabetes mellitus
DXA: Dual-energy X-ray absorptiometry
GED: General educational development
HbA1c: Hemoglobin A1c
HR: Hazard ratio
IQR: Interquartile range
LMR: Lymphocyte-to-monocyte ratio
LDL-C: Low-density lipoprotein cholesterol
NCHS: National Center For Health Statistics
NHANES: National Health and Nutrition Examination Survey
NDI: National Death Index
PIR: Poverty income ratio
ROC: Receiver operating characteristic
TC: Total cholesterol
US: United States.

## Supporting Information

Supporting Information 1 Table S1. Baseline characteristics of included participants according to survival and death. Abbreviations: AAC, abdominal aortic calcification; CHD, coronary heart disease; HbA1c, glycohemoglobin; HDL-C, high-density lipoprotein cholesterol; LDL-C, low-density lipoprotein cholesterol; LMR, lymphocyte-to-monocyte ratio; PIR, family poverty income ratio; WBC, white blood cell.

Supporting Information 2 Figure S1. The predictive ability of lymphocyte and monocyte alone for all-cause mortality in US adults with AAC. Time-dependent ROC curves (A) and time-dependent AUC values (B) (with 95% CI) of the lymphocytes alone for predicting all-cause mortality; and time-dependent ROC curves (C) and time-dependent AUC values (D) (with 95% CI) of the monocytes alone for predicting all-cause mortality. AAC, abdominal aortic calcification; AUC, area under the curve; CI, confidence interval; LMR, lymphocyte-to-monocyte ratio; ROC, receiver operating characteristic; US, United States.
