# Supplementary material for: Lymphocyte-to-Monocyte Ratio and All-Cause Mortality in Populations With Abdominal Aortic Calcification: A Prospective Cohort Study
Source: Mediators Inflamm. 2025 Jul 12;2025:9358261. doi: 10.1155/mi/9358261 (PMC12276054; doi:10.1155/mi/9358261)
Supplement: Supporting Information 1 — Table S1. Baseline characteristics of included participants according to survival and death. Abbreviations: AAC, abdominal aortic calcification; CHD, coronary heart disease; HbA1c, glycohemoglobin; HDL-C, high-density lipoprotein cholesterol; LDL-C, low-density lipoprotein cholesterol; LMR, lymphocyte-to-monocyte ratio; PIR, family poverty income ratio; WBC, white blood cell. [file 9358261.f1.docx]

**Table S1. Baseline characteristics of included participants according to survival and death**

| Variable Names | Survival | Death | *P* value |
| --- | --- | --- | --- |
| N | 743 | 145 |  |
| Age (year) | 63.11±11.69 | 73.67±8.64 | <0.01 |
| Sex (%) |  |  |  |
| Female | 368 (49.53) | 75 (51.72) | 0.69 |
| Male | 375 (50.47) | 70 (48.28) |  |
| Race (%) |  |  |  |
| Mexican American | 80 (10.77) | 11 (7.59) | <0.01 |
| Non-Hispanic Black | 116 (15.61) | 21 (14.48) |  |
| Non-Hispanic White | 378 (50.87) | 104 (71.72) |  |
| Other Hispanic | 67 (9.02) | 3 (2.07) |  |
| Other/multiracial | 102 (13.73) | 6 (4.14) |  |
| Education level (%) |  |  |  |
| 9-11th grade | 109 (14.67) | 13 (8.97) | 0.23 |
| >=College graduate | 179 (24.09) | 33 (22.76) |  |
| High school grad/GED | 179 (24.09) | 45 (31.03) |  |
| < 9th grade | 67 (9.02) | 15 (10.34) |  |
| Some college/AA degree | 209 (28.13) | 39 (26.90) |  |
| BMI | 27.79±4.7 | 27.02±5.27 | 0.08 |
| Waist size (cm) | 98.91±11.86 | 99.67±13.39 | 0.50 |
| PIR | 2.66±1.6 | 2.3±1.51 | 0.01 |
| Alocohol consumption (%) | 508(71.55) | 102(72.86) | 0.83 |
| Smoke status (%) |  |  |  |
| Current smoker | 160 (21.53) | 23 (15.86) | 0.03 |
| Former smoker | 236 (31.76) | 62 (42.76) |  |
| Never smoker | 347 (46.70) | 60 (41.38) |  |
| Platelet (10^3/uL) | 224.43±57.16 | 218.08±60.09 | 0.23 |
| Neutrophil (10^3/uL) | 4.26±1.59 | 4.69±1.81 | <0.01 |
| Lymphocyte (10^3/uL) | 2.1±0.78 | 1.94±1.02 | 0.03 |
| Monocyte (10^3/uL) | 0.59±0.19 | 0.7±0.31 | <0.01 |
| WBC (10^3/uL) | 7.21±2.02 | 7.6±2.31 | 0.04 |
| Glucose (mg/dL) | 115.28±26.75 | 114.63±26.41 | 0.79 |
| HbA1c (%) | 6.02±1.08 | 6.1±1.16 | 0.43 |
| Serum creatinine (mmol/L) | 86.14±60.62 | 105.7±75.7 | <0.01 |
| Serum uric acid (mmol/L) | 330.85±83.47 | 349.99±96.7 | 0.01 |
| Total cholesterol (mmol/L) | 4.99±1.08 | 4.75±1.3 | 0.02 |
| Triglycerides (mmol/L) | 1.43±0.61 | 1.35±0.52 | 0.19 |
| LDL-C (mmol/L) | 2.96±0.72 | 2.8±0.6 | 0.01 |
| HDL-C (mmol/L) | 1.36±0.39 | 1.41±0.47 | 0.15 |
| AAC total 24 score | 5±4.23 | 7.68±5.31 | <0.01 |
| Hypertension (%) | 426 (57.34) | 114 (78.62) | <0.01 |
| Diabetes (%) | 142 (19.11) | 39 (26.90) | 0.04 |
| Heart failure (%) | 28 (3.78) | 22 (15.17) | <0.01 |
| CHD (%) | 64 (8.61) | 28 (19.31) | <0.01 |
| Stroke (%) | 38 (5.11) | 22 (15.17) | <0.01 |
| LMR | 3.77±1.46 | 2.91±1.4 | <0.01 |

Abbreviation: AAC, Abdominal aortic calcification; CHD, Coronary heart disease; HbA1c, Glycohemoglobin; HDL-C, High-density lipoprotein cholesterol; LDL-C, Low-density lipoprotein cholesterol; LMR, Lymphocyte to monocyte ratio; PIR, family poverty income ratio; WBC, White blood cell.
