# Supplementary figures and images for: Lymphocyte-to-Monocyte Ratio and All-Cause Mortality in Populations With Abdominal Aortic Calcification: A Prospective Cohort Study
Source: Mediators Inflamm. 2025 Jul 12;2025:9358261. doi: 10.1155/mi/9358261 (PMC12276054; doi:10.1155/mi/9358261)

## Slide 1
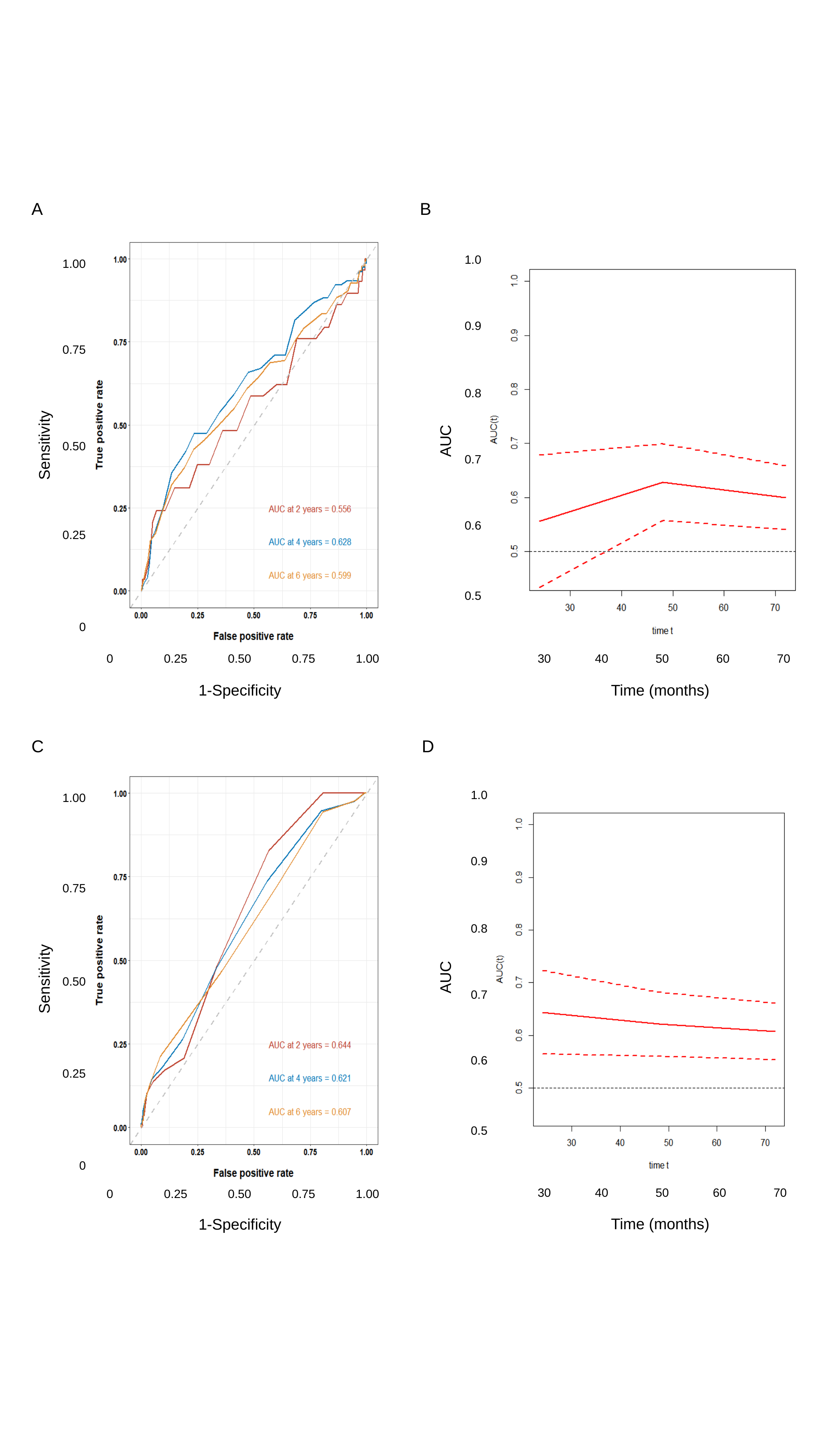

A B
1.0
0.9
0.8
0.7
0.6
0.5
1.00
0.75
0.50
0.25
0
Sensitivity
AUC
 0 0.25 0.50 0.75 1.00
30 40 50 60 70
1-Specificity
Time (months)
 C D
1.0
0.9
0.8
0.7
0.6
0.5
1.00
0.75
0.50
0.25
0
Sensitivity
AUC
30 40 50 60 70
 0 0.25 0.50 0.75 1.00
1-Specificity
Time (months)

Supplement: Supporting Information 2 — Figure S1. The predictive ability of lymphocyte and monocyte alone for all-cause mortality in US adults with AAC. Time-dependent ROC curves (A) and time-dependent AUC values (B) (with 95% CI) of the lymphocytes alone for predicting all-cause mortality; and time-dependent ROC curves (C) and time-dependent AUC values (D) (with 95% CI) of the monocytes alone for predicting all-cause mortality. AAC, abdominal aortic calcification; AUC, area under the curve; CI, confidence interval; LMR, lymphocyte-to-monocyte ratio; ROC, receiver operating characteristic; US, United States. [file 9358261.f2.pptx]
